# Correlation of Color Doppler with Multidetector CT Angiography Findings in Carotid Artery Stenosis

**DOI:** 10.1100/tsw.2010.170

**Published:** 2010-09-14

**Authors:** Živorad N. Savic, Lazar B. Davidovic, Dragan Ž. Sagic, Milan D. Brajovic, Srdjan S. Popovic

**Affiliations:** ^1^ Institute of Radiology, Clinical Center of Belgrade, Serbia; ^2^ Center for Cardiovascular Diseases, Clinical Center of Belgrade, Serbia; ^3^ Center for Cardiovascular Diseases Dedinje, Serbia; ^4^ Center for Cardiovascular Diseases Zvezdara, Serbia; ^5^ Institute of Endocrinology, Diabetes and Metabolic Diseases, Clinical Center of Belgrade, Serbia

**Keywords:** internal carotid artery, multidetector CT angiography, Color Doppler

## Abstract

The aim of this paper was to examine the correlation between the Color Doppler ultrasound (CD-US) and multidetector CT angiography (MDCTA) diagnostic methods, and to define the degree and extent of stenosis in patients with internal carotid artery stenosis. This was a cross-sectional study with a consecutive series of patients. All US examinations were always carried out by the same physician-angiologist, while all CT examinations were always carried out by the same physician-radiologist. Both worked independently from each other. The stenosis area was measured at the narrowest point by NASCET criteria for US/CT. Peak systolic velocity (PSV) over 210 cm/sec and end diastolic velocity (EDV) over 110 cm/sec criteria were applied for stenoses with lumen narrowed over 70%, while PSV under 130 cm/sec and EDV under 100 cm/sec criteria were applied for those with lumen narrowed under 70%. A total of 124 carotid arteries were observed; namely, 89 narrowed and 68 surgically treated. All patients were reviewed by US and then by MDCTA; patients with 70–99% stenosis underwent surgery. The correlation coefficient between stenosis degree measured by US and MDCTA was 0.922; *p* < 0.01. The average difference between US and MDCTA diagnostic methods was 3% (Z = -1.438, *p* > 0.05). The US and CT matching level for stenoses from 70 to 99% was very high (κ = 0.778, *p* < 0.01). In conclusion, there is a highly significant statistical correlation among both diagnostic methods when measuring stenosis degree and extent. US is more dependent on the physician, while MDCTA is more objective and independent from the physician. We think it would be appropriate to undertake an MDCTA exam for those patients who are candidates for carotid endarterectomy.

## INTRODUCTION

Internal carotid artery stenosis is a major cause of stroke (over 700,000 cases per annum in the U.S.[1]. Of patients reviewed by noninvasive methods, 20–25% suffered a stroke caused by carotid artery embolism[2]. In patients with transient ischemic attacks (TIAs), it was necessary to examine their internal carotid arteries (ICAs) because the stroke risk after TIA significantly increases[3,4,5]. The authors analyzed the correlation between Color Doppler ultrasound (CD-US) and 64-multidetector CT angiography (MDCTA) findings, and the degree and extent of ICA stenoses. Precise and reliable findings are important in order to plan for surgical intervention (revascularization)[6]

## MATERIALS AND METHODS

This was a cross-sectional study on a consecutive series of patients. A total of 124 carotid arteries were observed in 62 patients. Narrowing of carotid artery stenosis was found in 89 samples and 68 surgical interventions were performed. ICA unilateral stenosis was found in 35 (56.4%) patients, while bilateral stenosis was found in 27 (43.6%) patients. All patients were reviewed by US and then by 64-MDCTA. Patients with 70–99% stenosis as measured by the NASCET method [formula 100x (A-B)/A**,** US and criteria for CT] underwent surgery. Length and extent of the stenosis area were measured. US examinations were performed by an angiologist and MDCTA examinations by a radiologist. All US examinations were always carried out by the same physician-angiologist, while all CT examinations were always carried out by the same physician-radiologist. Both worked independently from each other. Patients were aged 35–81 (average age: 64.7 ± 11.5); 42 male (67.7%) and 20 female (32.3%). After clinical (neurological) examination, all patients sent by the neurologist because of TIA, reversible ischemic neurologic deficit (RIND), prolonged ischemic neurological deficit (PIND), cerebrovascular insults (CVI), or amaurosis fugax (these are including criteria) were preoperatively submitted to CD-US examination of the degree and extent of ICA. Patients were classified into three groups by degree of stenosis and according to NASCET criteria (measuring intra- and poststenotic segments): stenosis up to 69%, stenosis from 70 to 99%, stenosis of 100% (occlusion). There were findings on ICA with stenoses below 69%, which were not taken into further consideration because surgery is not carried out in patients with such stenoses. According to the excluding criteria, the following were excluded: patients previously operated on for carotid disease, those with already verified thrombosis in the left ventricle and/or auricle, patients with verified brain tumors, patients with intracerebral hemorrhage, patients allergic to contrast agents, patients with very damaged renal function, and patients with hyperthyreosis.

All patients with 70–99% stenosis underwent surgery. The initial evaluation was performed by US (Siemens Acuson Antares) with multifrequent linear probe of 5–10 MHz. The evaluation was then repeated using the 64-MDCTA (GE Light Speed VCT 64), with a mandatory use of an automatic injector (contrast flow not less than 4.5 ml/sec, amount 1.2–1.5 ml/kg body mass of patient). CT examination understood MDCTA in supra-aortic vessel level, including the carotid siphon. Postprocessing analysis included the analysis of arteries in the “SSD mode view” vessel and classic cross-sections, in order to specify the width and length of lumen stenosis in the ICA. The authors used descriptive statistical methods (measures of central tendency [AS and MED] and dispersion measures [SD, I]) and analytical statistical methods (Mann-Whitney U-test, ANOVA test, Spearman’s correlation analysis, κ coefficient matching, Cronbach’s α). Results were displayed graphically with diagrams and ternary scatter graphs.

## RESULTS

As seen in Table 1, arithmetic means differ by 3% and medians are identical. There are no statistically significant differences in the evaluation of stenosis of the two diagnostic methods (US and CT), (Mann-Whitney U, Z = –1.438, $p > 0.05$).

**TABLE 1**
**Mean, Median, Minimum and Maximum Measured Stenosis**

| | N | Mean | Median | Minimum | Maximum | Range | Std. Deviation |
|---|---|---|---|---|---|---|---|
| US % stenosis | 89 | 82.61 | 85.00 | 22 | 100 | 78 | 14.345 |
| CT % stenosis | 89 | 79.62 | 85.00 | 25 | 100 | 75 | 15.406 |

Almost half of the samples (38/64) had the same US/CT stenosis values, while 24/64 differed by only 5%. Other frequencies were negligible (2/64).

Fig. 1 and Table 2 show each individual stenosis obtained by US compared with CT values. US values coincide with CT values. There is a statistically strong correlation between US and CT ($p < 0.01$), and very high ($r = 0.922$) correlation of these two methods.

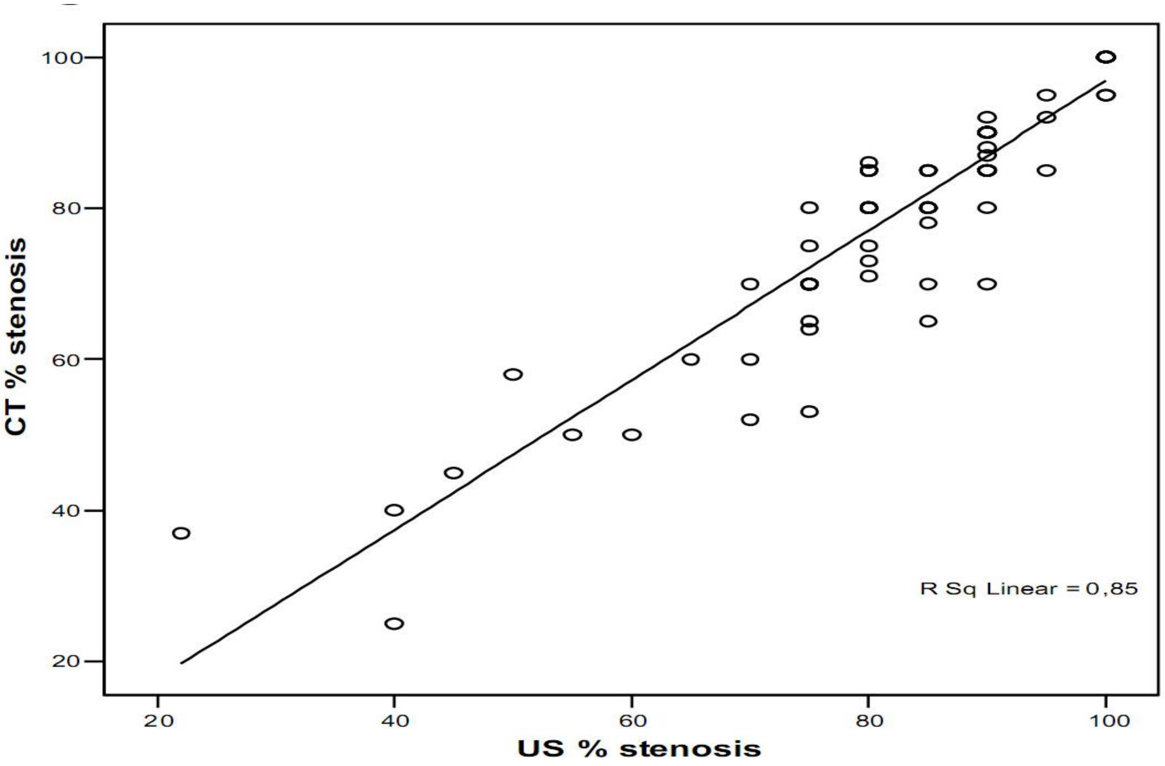

**FIGURE 1.** Superposition method, diagnostic US/CT in measuring the degree of stenosis.

Comparison of US and CT findings: Sp = 0.57, Sn = 1, PPV= 0.926, NPV = 1 (shown in Fig. 2). If the degree of stenosis is translated into categories: stenosis up to 69%, stenosis 70–99%, occlusion (100%), these two diagnostic methods match by cross-tabulation.

Table 3 shows that stenoses 70–99% and occlusions almost match in both diagnostic methods, whereas stenoses up to 69% slightly differ. Measures of direction and symmetry were: Somer's D = 0.820, $p < 0.01$; Spearman's correlation = 0.832, $p < 0.01$. Matching measures: $\kappa = 0.778$, $p < 0.01$. There is a strong connection between US and CT diagnostic methods, not accidental, because significance is always under 0.01.

**TABLE 2**
**Superposition Method, Diagnostic US/CT in Measuring the Degree of Stenosis (Individual Values)**

| | | Frequency | Valid Percent | Cumulative Percent |
|---|---|---|---|---|
| Valid | –15 | 1 | 1.1 | 1.1 |
| | –8 | 1 | 1.1 | 2.2 |
| | –6 | 1 | 1.1 | 3.4 |
| | –5 | 5 | 5.6 | 9.0 |
| | –2 | 1 | 1.1 | 10.1 |
| | 0 | 38 | 42.7 | 52.8 |
| | 2 | 1 | 1.1 | 53.9 |
| | 3 | 2 | 2.2 | 56.2 |
| | 5 | 24 | 27.0 | 83.1 |
| | 7 | 2 | 2.2 | 85.4 |
| | 9 | 1 | 1.1 | 86.5 |
| | 10 | 5 | 5.6 | 92.1 |
| | 11 | 1 | 1.1 | 93.3 |
| | 15 | 2 | 2.2 | 95.5 |
| | 18 | 1 | 1.1 | 96.6 |
| | 20 | 2 | 2.2 | 98.9 |
| | 22 | 1 | 1.1 | 100.0 |
| | Total | 89 | 100.0 | |

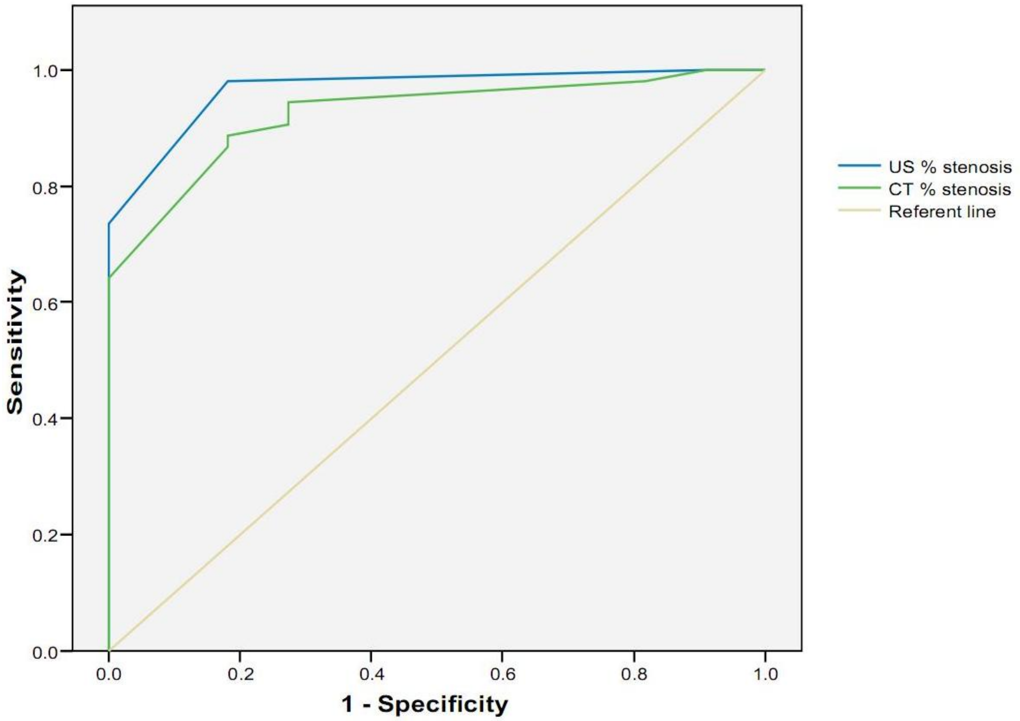

**FIGURE 2.** ROC curve of the observation stenosis, comparing with US/CT (%).

**TABLE 3**
**Stenoses Divided into Three Categories and Their Agreement with US/CT**

| | | CT Diagnostic | | | Total |
|---|---|---|---|---|---|
| | | Stenosis Up to 69% | Stenosis 70–99% | Occlusion | |
| US diagnostic | Stenosis up to 69% | 8 | 0 | 0 | 8 |
| | Stenosis 70–99% | 6 | 63 | 0 | 69 |
| | Occlusion | 0 | 2 | 10 | 12 |
| Total | | 14 | 65 | 10 | 89 |

The average difference between the US and CT was not statistically significant and amounts to 0.061.

Tables 4 and 5 and Fig. 3 show that US values coincide with CT values. Correlation is statistically highly significant and very high (r = 0.934, $p < 0.01$).

**TABLE 4**
**US/CT Extent of Stenosis**

| | N | Mean | Median | Minimum | Maximum | Range | Std. Deviation |
|---|---|---|---|---|---|---|---|
| US length of stenosis | 81 | 21.91 | 20.00 | 6 | 50 | 44 | 8.497 |
| CT length of stenosis | 81 | 21.74 | 20.00 | 6 | 50 | 44 | 8.414 |

**TABLE 5**
**Difference in Stenosis Extents, US and CT Individually**

| | | Frequency | Percent | Valid Percent | Cumulative Percent |
|---|---|---|---|---|---|
| Valid | –6.00 | 1 | 1.1 | 1.2 | 1.2 |
| | –5.00 | 6 | 6.8 | 7.4 | 8.6 |
| | –4.00 | 1 | 1.1 | 1.2 | 9.9 |
| | –3.00 | 4 | 4.5 | 4.9 | 14.8 |
| | –2.00 | 6 | 6.8 | 7.4 | 22.2 |
| | –1.00 | 4 | 4.5 | 4.9 | 27.2 |
| | .00 | 41 | 46.6 | 50.6 | 77.8 |
| | 1.00 | 2 | 2.3 | 2.5 | 80.2 |
| | 2.00 | 2 | 2.3 | 2.5 | 82.7 |
| | 3.00 | 2 | 2.3 | 2.5 | 85.2 |
| | 4.00 | 1 | 1.1 | 1.2 | 86.4 |
| | 5.00 | 9 | 10.2 | 11.1 | 97.5 |
| | 10.00 | 1 | 1.1 | 1.2 | 98.8 |
| | 11.00 | 1 | 1.1 | 1.2 | 100.0 |
| | Total | 81 | 92.0 | 100.0 | |

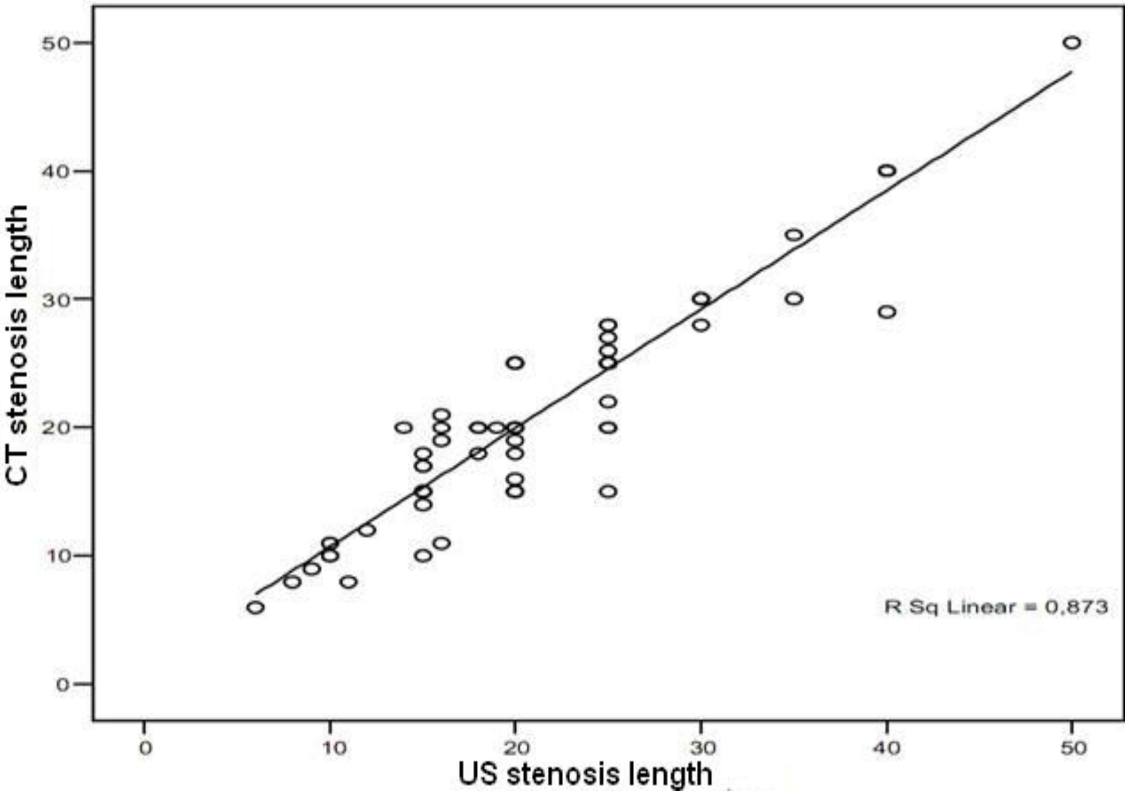

**FIGURE 3.** Superposition method, diagnostic US/CT in measuring the extent of stenosis.

## DISCUSSION

US, which involves the use of Doppler and Color Doppler techniques[6,7,8], is often the only diagnostic method used to determine the degree and extent of carotid artery stenosis. US examination is frequently used in patients with high-degree stenosis (in order to prevent cerebral ischemia and stroke) and with artery occlusion (to avoid less successful or unsuccessful surgery). US is not applicable as a reliable diagnostic method in patients with short neck, obesity, presence of edema and scar after irradiation treatment, when the bifurcation set is high, or when changes in the distal part of the artery are not available to US review. Ultrasonic diagnosis still largely depends on the skill of the examiner[7,8,9]. Annular calcification and thick artery walls also cause difficulties in the US diagnosis. In such cases, other diagnostic methods may be performed: subtraction digital angiography (DSA), MDCTA, or magnetic resonance angiography (MRA)[10,11,12,13,14,15,16,17,18,19,20]. Agreement between multidetector row computed tomography (CT), MDCTA, and CD-US in measuring carotid artery wall thickness (CAWT) and the intima-media thickness (IMT)[21] has been applied recently. Comparing the results obtained individually, discrepancy of US and MDCTA findings with intraoperative findings (IOF) was observed. When the degree of stenosis was translated into categories (stenosis up to 69%, stenosis 70–99%, occlusion 100%), there was little difference between US/CT and IOF in the 69% stenosis category. The extent of stenosis obtained in IOF is a subjective evaluation of the surgeon because he also included the IMT (under 2 mm) and parts of healthy vessels at both ends of the stenotic vessel, while US and CT measured plaque extent only.

## CONCLUSION

The results of our study suggest a significant agreement between MDCTA and CD-US in measurement of the degree and extent of stenosis. CD-US is the most widely accessible test in most centers worldwide and, therefore, it seems reasonable to use it as first-line investigation. However, because of its relative insensitivity in detecting some plaque characteristics of risk and relative insensitivity in stenosis quantification, it would be unwise to rely on the CD-US exam alone. We think it would be appropriate to undertake the MDCTA exam for those patients who are candidates for carotid endarterectomy because there were cases where MDCTA was advantageous, i.e., highly placed bifurcation, short neck, changes in ICA siphon, large scars in neck area, postirradiation therapies, massive calcifications of blood vessel walls. The accuracy of the US examination is strongly dependent on the skills of the examiner.
